# Superpose3D: A Local Structural Comparison Program That Allows for User-Defined Structure Representations

**DOI:** 10.1371/journal.pone.0011988

**Published:** 2010-08-05

**Authors:** Pier Federico Gherardini, Gabriele Ausiello, Manuela Helmer-Citterich

**Affiliations:** Centre for Molecular Bioinformatics, Department of Biology, University of Rome “Tor Vergata”, Rome, Italy; Griffith University, Australia

## Abstract

Local structural comparison methods can be used to find structural similarities involving functional protein patches such as enzyme active sites and ligand binding sites. The outcome of such analyses is critically dependent on the representation used to describe the structure. Indeed different categories of functional sites may require the comparison program to focus on different characteristics of the protein residues. We have therefore developed superpose3D, a novel structural comparison software that lets users specify, with a powerful and flexible syntax, the structure description most suited to the requirements of their analysis. Input proteins are processed according to the user's directives and the program identifies sets of residues (or groups of atoms) that have a similar 3D position in the two structures. The advantages of using such a general purpose program are demonstrated with several examples. These test cases show that no single representation is appropriate for every analysis, hence the usefulness of having a flexible program that can be tailored to different needs. Moreover we also discuss how to interpret the results of a database screening where a known structural motif is searched against a large ensemble of structures. The software is written in C++ and is released under the open source GPL license. Superpose3D does not require any external library, runs on Linux, Mac OSX, Windows and is available at http://cbm.bio.uniroma2.it/superpose3D.

## Introduction

The increasing number of structures available as a result of structural genomic initiatives has generated great interest in the development of structure-based function prediction methods [1], [2]. Similar to sequence analysis the most straightforward approach is to compare the protein to be characterized with a set of proteins of known function. Global structural comparison methods, such as Dali [3], Vast [4] SSM [5] and CE [6], can be used to identify remote homology relationships that defy traditional sequence analysis.

In addition, since the function of a protein usually depends on the identity and location of a small number of residues, local structural comparison methods (reviewed in [1]) represent the ideal tool to focus the comparative analysis on the residues which are critical to function. Therefore one can compare a protein of unknown function with a set of well-characterized structures in order to check whether there are local similarities involving the known functional patches. Alternatively, from the analysis of a number of structures sharing some property, it is possible to derive a structural template encoding the function-determining residues, and use that to screen the proteins of interest.

The local comparison problem comprises two different tasks:

- finding a suitable representation for the protein structure
- searching for the correspondence between the descriptors used that is optimal according to some criteria (e.g. length, RMSD, or a combination of both).

As we will show, the type of representation used can greatly influence the kind of results that are obtained by the application of these methods. Indeed different functional sites may require a residue description focused on different physicochemical properties.

In terms of search strategy three approaches are commonly used: recursive branch and bound algorithms, subgraph isomorphism and geometric hashing. The first two algorithmic strategies are equivalent in practice. A recursive branch and bound algorithm is used by RIGOR/SPASM [7], Query3d [8] and PINTS [9]. Methods based on subgraph isomorphism include ASSAM [10], CavBase [11] and eF-Site [12]. Methods relying on geometric hashing include C-alpha Match [13], Prospect [14], SiteEngine [15] and ProteMiner-SSM [16].

However the two tasks of representing the structure and searching for correspondences can be decoupled. Indeed, once a structure representation has been calculated according to the specific method used by the program, however complex this step may be, the problem simply becomes that of finding a correspondence between two sets of descriptors in space. We present here a novel program that leverages this observation. This program is called superpose3D and is available under the open source GPL license at http://cbm.bio.uniroma2.it/superpose3D. Superpose3D allows users to flexibly specify the way that residues are to be represented during the computation and the pairing rules.

To the best of our knowledge the only downloadable, open-source methods for local structural comparison are RIGOR/SPASM and PINTS. RIGOR/SPASM allows the user to specify the residue substitutions. However, in terms of structure representation, the only option is whether to use the CA, the geometric centroid of the side chain or both.

The residue definition syntax of PINTS is much more flexible. Users are required to assign arbitrary types to different atoms. Atoms of the same type are part of the same equivalency group and therefore can be matched with each other. Therefore it is not possible to specify that atoms A–B of residue X must match atoms C–D of residue Y and have to be paired as A;C B;D. In other words it is not possible to specify constraints that involve more than one equivalence group at the same time. Moreover when multiple atoms are selected for the same residue PINTS always uses the geometric centroid.

In this work we describe superpose3D and the syntax used to specify different residue descriptions. We will also discuss several examples that highlight the advantages of using three types of structure description with varying levels of detail. These examples underscore the importance of using a residue representation that is tailored to the analysis at hand.

## Methods

### Design and implementation

Superpose3D was written in C++ and does not require any external library. The software runs on Linux, Mac OSX and Windows. The program needs as input a file specifying which structures are to be compared, and eventually which residues in each chain, and another file containing the residue representation to be used together with a number of additional parameters.

The residue description syntax is built around the notion of “pseudoatom”. A pseudoatom is a point used by the program to represent a residue or part of it. The number of pseudatoms that are used to represent each residue clearly influence the level of detail of the representation. Such points may correspond to an actual protein atom, e.g. the CA of a residue. However this is not necessarily the case. For instance one could define a pseudoatom corresponding to the geometric centroid of the side chain or any other set of atoms. This allows to include information about the location of specific chemical groups without necessarily increasing the complexity of the representation. Moreover when groups are represented this way there is more room for positional variation as it is not required for all the atoms to align exactly.

The syntax of superpose3D allows users to define the pseudoatoms that represent each residue (including modified amino acids) by referring to the standard PDB atom naming convention. Once pseudoatoms have been defined it is necessary to describe the rules with which they can be paired to form a structural match.

The software package includes three residue definition files corresponding to the representation used in previous works [8], [9], [11] so that users need not develop a specific structure representation before being able to use the program. Therefore users can start from structure descriptions already used in the literature, eventually modifying them as they see fit.

### Specifying residue representations and equivalences

**Figure 1:**
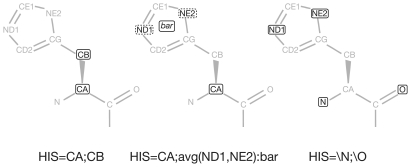
Alternative residue representations. Three alternative ways to represent histidine, along with the corresponding syntax used by superpose3D. The atoms are named according to the PDB standard. The “avg(ND1,ND2):bar” statement (middle) defines a pseudoatom named “bar” whose coordinates correspond to the geometric centroid of the ND1 and NE2 atoms. The “\N;\O” statement (right) specifies that all the atoms that contain an “N” or “O” in their names should be included in the representation.

Figure 1 displays three alternative ways to represent histidine together with the syntax used by the program. Each residue is specified in a line starting with the keyword “def”. The format is as follows:
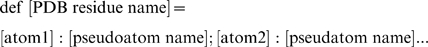



For instance the following line specifies that serine should be represented by two points, corresponding to the CA and CB atoms.
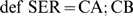



It is also possible to define a “pseudoatom” as the geometric centroid of a user-specified list of PDB atoms.

The syntax is as follows:
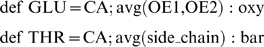



Once the user has defined the points that the program should use to represent residues a list of equivalences has to be provided. These statements specify which residues are allowed to match and which points should be used for the superimposition.

The syntax is as follows (the .[atom] part is optional)
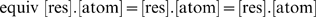
For instance the statement
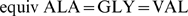
instructs the program that the residues ALA, GLY and VAL are equivalent and can be matched with each other. By default the points that are used to represent the residues will be paired in the same order in which they appear in the definition. However it is also possible to specify equivalences between specific residue fragments. For instance given the definitions above one could write the following equivalence:
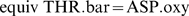



It is also possible to specify multiple atoms together, e.g.:
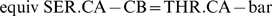
Once again atoms will be paired according to the order in which they are written.

Furthermore wildcards can be used to specify groups of residues or atoms. Two types of wildcards are available asterisk ‘*’ and backslash ‘\’. ‘*’ is used in residue names and it means ‘Any residue that does not match a more specific definition’. ‘\’ is used in atom names with the meaning ‘Any atom whose name contains the string following the backslash’. For instance the following lines:
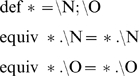
mean that all the residues should be represented with all their nitrogen and oxygen atoms and that only atoms of the same type are allowed to match.

Users can also specify whether they want all the constituent atoms of a residue to be treated independently, so that two fragments of a single residue can be matched with two different residues, or not. This is an important difference with PINTS because it allows to increase the level of detail without necessarily increasing the computational cost. Indeed as long as two residues are matched as single entities the number of points that are used for their representation does not influence the running time of the algorithm.

### Search algorithm

The structural comparison algorithm uses a branch & bound strategy to find the largest subset of pseudoatoms between two protein structures that can be superimposed under a given RMSD threshold, irrespective of their position along the sequence. During this search only residues (or single pseudoatoms) which have been defined as equivalent will be paired. The algorithm starts by creating all the possible equivalences between single elements. These matches are then extended using a recursive, depth-first, search procedure. The matches are evaluated and kept if their constituent elements can be superimposed with an RMSD lower than the threshold currently in use. The optimal superimposition between two sets of points is calculated using the Quaternion method [17]. When the exploration is finished the algorithm returns all the matches of maximum length.

### Running time and complexity

The complexity of the procedure is exponential in the size of the probe and target chains. In practice the software is extremely fast as long as the search tree is pruned early. Consequently the running time increases as the RMSD gets higher or the number of possible correspondences between residues is increased. For the same reason, similarly to other methods [9], superpose3D is not suited to the analysis of complete structures if they are obviously homologous. Indeed such cases should be analysed with global comparison programs. However using an appropriate RMSD threshold and given the average size of protein chains, meaningful results can usually be obtained rather quickly. For instance comparing a ∼300 residues chain with the non-redundant ASTRAL compendium (10563 chains) [18], representing each residue with the Cα and the centroid of the side chain and using 0.7 as the RMSD threshold, takes ∼8 minutes to load all the structures and ∼12 minutes to run the comparison on a 2.4 GHz Intel Core 2 processor.

## Results and Discussion

In order to highlight the advantages of using different residue representations we present detailed examples of the results that can be obtained with three different structure descriptions of increasing complexity. We choose to focus on binding sites as the position of the ligands can readily be used to assess the functional significance of a structural similarity. The examples presented in the following paragraphs are discussed with the specific aim of showing how different representations affect the outcome of the analysis.

### Coarse representation

We first used an extremely coarse representation that considers only the position of the Cα and permits any residue substitution. This is the aminoacid description that is often used by fold comparison algorithms. However this representation can also be useful, as the following examples show, when comparing binding sites. We included two examples that would likely be missed by fold comparison algorithms as they involve proteins with different overall structures.

**Figure 2:**
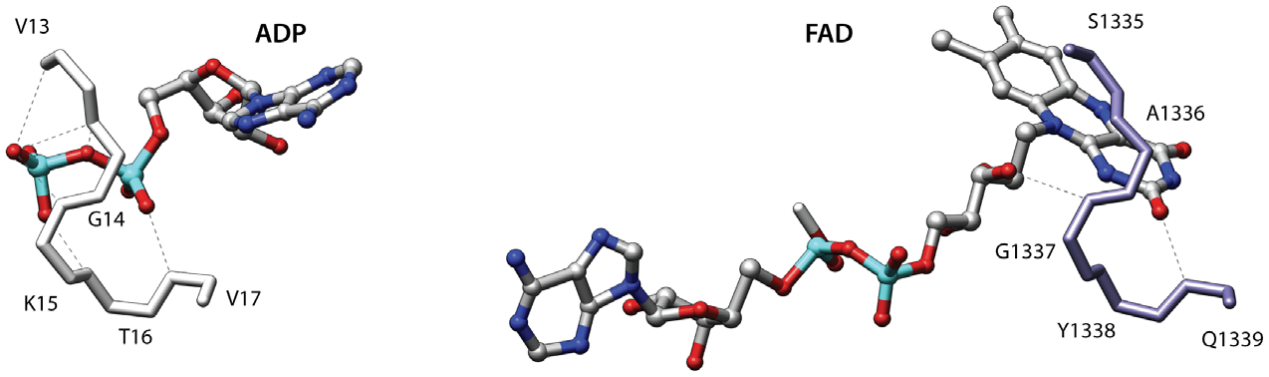
Comparison of two anion binding loops. The loop on the left binds phosphate while the right one binds the O2 and O3′ of the riboflavine moiety of FAD. Left: dethiobiotin synthetase from *Escherichia coli* (1dak); right: D-amino acid oxidase from the yeast *Rhodotorula gracilis* (1c0i). In this figure and in the following ones protein residues are represented as sticks and ligands as ball and sticks. Moreover ligand names are written in bold.

Figure 2 displays the similarity between dethiobiotin synthetase from *Escherichia coli* (1dak) [19] and D-amino acid oxidase from the yeast *Rhodotorula gracilis* (1c0i) [20]. When comparing the binding sites of these two proteins superpose3D finds a structural match involving the Cα of eight different residues which can be superimposed with an RMSD of 0.66 Å. Dethibiotin synthetase belongs to the extensive group of nucleoside triphosphate hydrolases containing the characteristic phosphate-binding P-loop. Conversely the D-amino acid oxidase belongs to the “Nucleotide-binding domain” fold, which is a member of the large group of Rossmann-like folds. This protein binds the O2 and O3′ of the riboflavine moiety using a loop which is very similar to the P-loop of Dethibiotin synthetase. These compact loops often bind anions with hydrogen bonds to main chain atoms and have been termed “nests” [21]. Given this mode of binding the identity of the residues and the position of the side chain is not important in this case. Accordingly four of the five residues that comprise the loops have negative substitution scores in a BLOSUM62 matrix.

**Figure 3:**
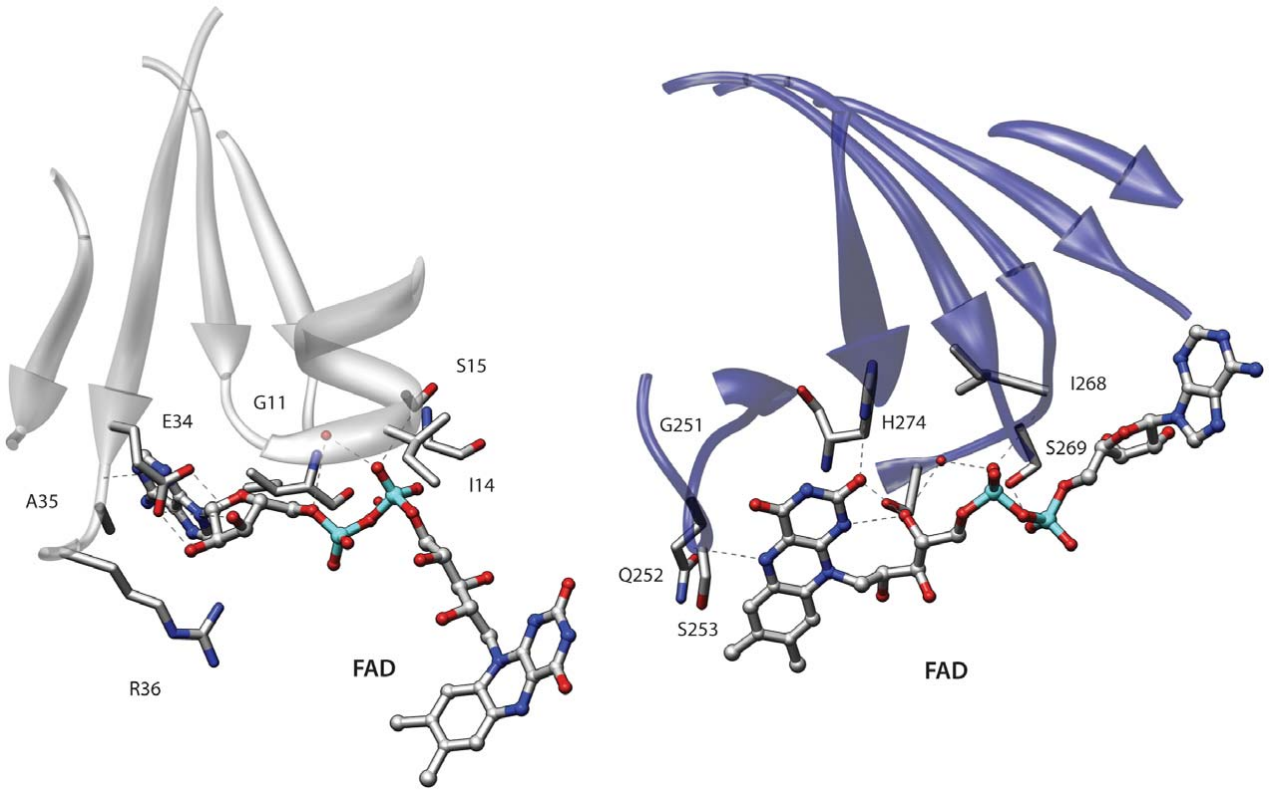
Comparison of two FAD binding sites. The two binding pockets belong to proteins of different Rossmann-like folds. Left: human monoamine oxidase B (2v61); right: Electron transfer flavoprotein from *Methylophilus methylotrophus* (3clt). The central β-sheets that characterize these structures are shown in the picture but only the binding site residues were used in the comparison.

Another interesting example of similarity involves the human monoamine oxidase B (2v61) [22] and an electron transfer flavoprotein from *Methylophilus methylotrophus* (3clt). These proteins belong to two different folds of the Rossmann-like group and probably share a remote ancestor [23]. Even though these folds are related the fold comparison program DaliLite [3] finds an alignment with a non-significant score that fails to correctly superpose the ligands. Conversely superpose3D finds a structural match comprising eight residues with an RMSD of 0.64 Å. Figure 3 shows the alignment between the binding pockets. Once again we used a description that only considers the Cα of each residue. The program correctly identifies the similarity between the two binding sites. Interestingly in this case using a representation that includes side-chain information resulted in a different yet still meaningful match (see below).

### Including side-chain information

The easiest way to include side-chain information is to add to the Cα a point corresponding to the geometric centroid of the side-chain atoms. In the following examples we also restricted residue substitutions by only allowing matches between residues with a substitution score of at least -1 in a BLOSUM62 matrix. This representation allows to include side-chain information without increasing the computational cost, because residues are still paired as single entities, i.e. the points used to represent them are not treated independently (see Methods).

Using this representation on the same proteins depicted in Figure 3 resulted in the identification of a different similarity. As mentioned the two proteins involved belong to the well-known group of Rossmann-like folds. This structure is characterized by a central β-sheet in which α-helices connect the strands together. One of these βαβ units contains a characteristic glycine-rich phosphate binding loop [24].

Interestingly the β-sheets of the monoamine oxidase and the electron transfer flavoprotein have a permuted structure. In the first protein the phosphate binding loop occurs in the N-terminal βαβ unit of the sheet. Conversely the phosphate binding loop of the electron transfer flavoprotein is located in the second βαβ unit. The two β-sheets also have a different twist. Indeed if one superimposes the two β-sheets in an optimal way the ligand binding sites end up in opposite positions. The match shown in Figure 3 comprises eight residues, captures the overall similarity between these two binding sites and places the phosphate binding elements in similar positions. However in terms of the overall structures the β-sheets are placed one in front of the other.

**Figure 4:**
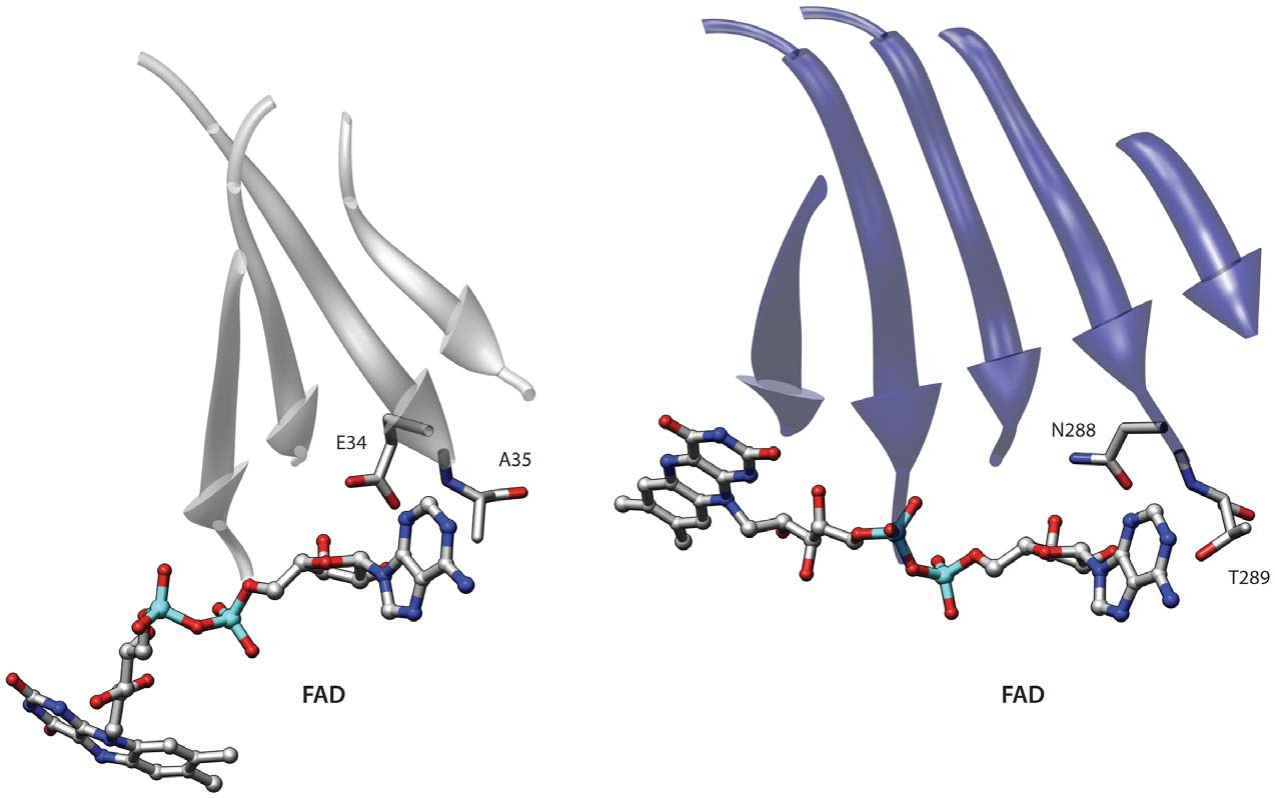
Comparison of two FAD binding sites including side chain information. The same binding pockets depicted in Figure 3 were compared using a description that includes side-chain information. The residues comprising the β-sheets were not used in the comparison. See text for details.

If we include side-chain information and re-run the comparison we obtain an interesting albeit much smaller similarity. Indeed as shown in Figure 4 these proteins also have an identical adenine binding motif, which was also described by Denessiouk and Johnson [25]. This second alignment is also informative because it highlights a similar adenine recognition site in these proteins. In this alignment the central β-sheets are closer in space, even though an alignment that simultaneously superimposes both the central sheets and the binding sites is not possible due to the above-mentioned permutation. Therefore these two proteins provide an interesting example of the results that can obtained using different levels of detail in the representation.

**Figure 5:**
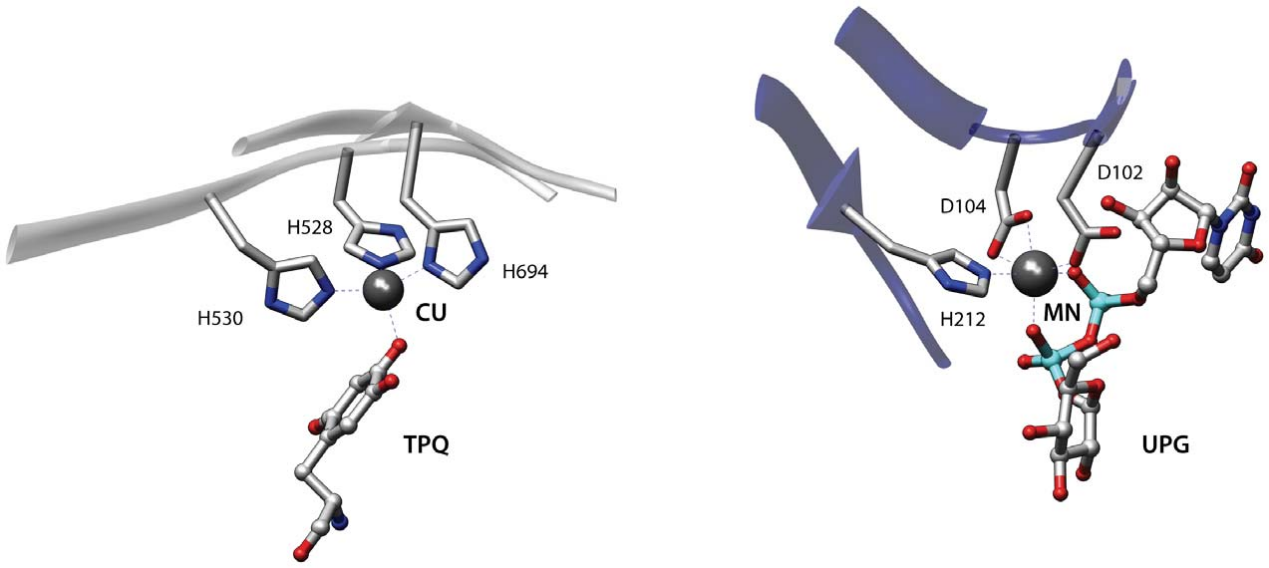
Comparison of two metal coordination sites. The proeins involved are Lysyl oxidase from *Pichia pastoris* (1w7c, left) and rabbit glycogenin-1 (1ll2, right).

Figure 5 displays a metal coordination site shared by Lysyl oxidase from *Pichia pastoris* (1w7c) and rabbit glycogenin-1 (1ll2) that belong to the Supersandwich and Nucleotide-diphospho-sugar transferases folds respectively. The structural match includes three residues with an RMSD of 0.48 Å. This representation is well-suited to a case like this since metal binding sites usually have a fixed geometry and are composed of specific aminoacid types. However, as the following paragraphs show, there are more subtle examples of similarity that require a more detailed representation.

### Detailed representation focused on specific chemical groups

The third representation we used is modelled after the one introduced by Schmitt *et al.* [11], modified not to include matches between main chain atoms. This representation is focused on the physicochemical properties of specific side-chain groups. An important difference between this description and the other ones we used is that each point is treated as a single independent entity, therefore the same residue can match with more than one residue, using different atoms.

**Figure 6:**
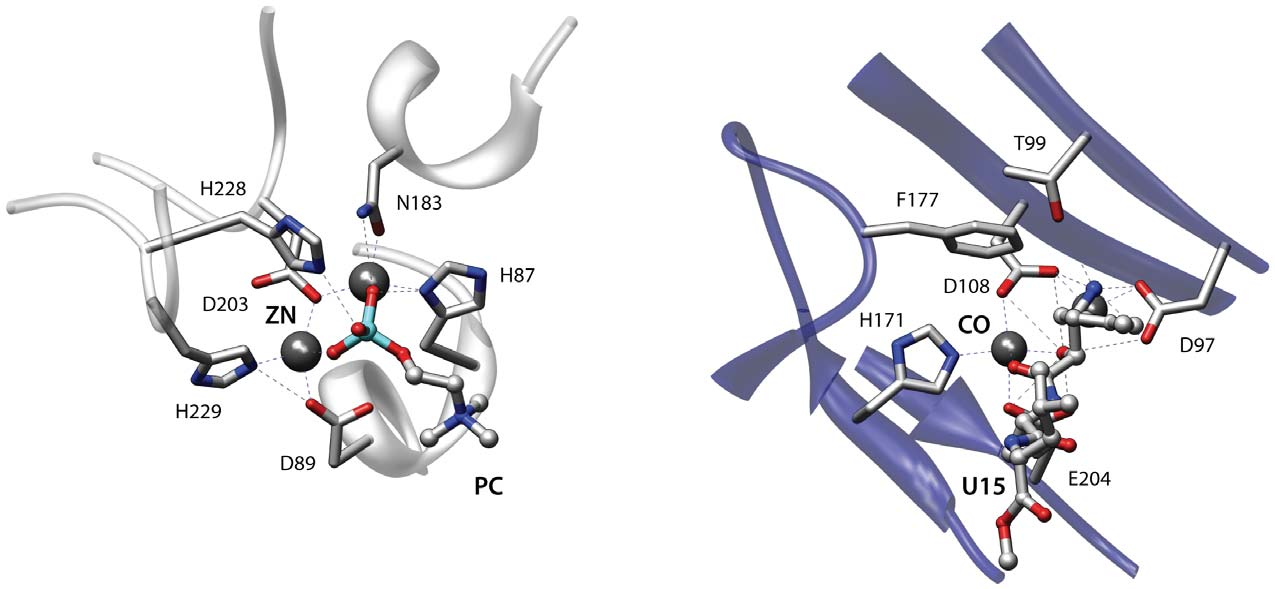
Two enzymes with similar substrate binding sites. The figure depicts the active sites of the teichoic acid phosphorylcholine esterase Pce from *Streptococcus pneumoniae* (2bib, left) and a methionine aminopeptidase from *Escherichia coli* (2gg8, right). These unrelated enzymes use similar mechanisms and have analogous binding modes for the substrate (left) and an inhibitor (right).

A metal-dependent mechanism is often involved in the hydrolysis of peptide and ester bonds [26] and examples of convergent evolution between lactamases and metalloaminopeptidases have already been reported [27]. Figure 6 shows an alignment of the active sites of the teichoic acid phosphorylcholine esterase Pce from *Streptococcus pneumoniae* (2bib) [28] and of a methionine aminopeptidase from *Escherichia coli* (2gg8) [29]. For both enzymes a mechanism has been proposed whereby the two metal ions in the active site activate a water molecule for nucleophilic attack and participate in the stabilization of the resulting tetrahedral intermediate [28], [30]. The two proteins are unrelated and belong to different SCOP [31] folds. When comparing the binding sites of these proteins superpose3D identified a match comprising eight pseudoatoms belonging to six residues with an RMSD of 0.68 Å. Interestingly the phosphorylcholine esterase is complexed with its phosphocholine substrate while the methionine aminopeptidase is bound to an aminoacidic inhibitor. The algorithm therefore succeeded both in identifying a similarity between two unrelated enzymes that share similar mechanisms and also in highlighting the similar binding modes of the substrate and an inhibitor. The representation focused on chemical groups was absolutely necessary to identify the structural similarity. Indeed the metal binding residues Asp97, Asp108, His171 of the aminopeptidase (2gg8) and His87, Asp203, His229 of the phosphorylcholine esterase (2bib) only have the chemical groups that are involved in the interaction superimposed while the remaining atoms occupy different spatial positions.

**Figure 7:**
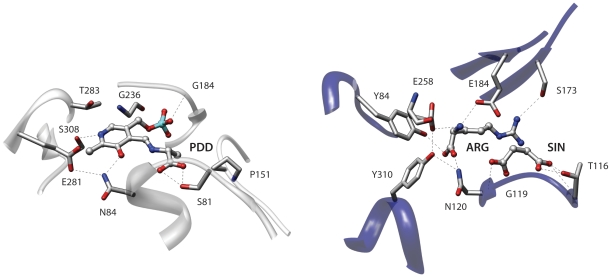
Comparison of two enzymes with similar substrate binding sites. The proteins involved are Serine racemase from *Schizosaccharomyces pombe* (2zpu, left) and Argininosuccinate synthetase from *Thermus thermophilus* (1kor, right).

**Figure 8:**
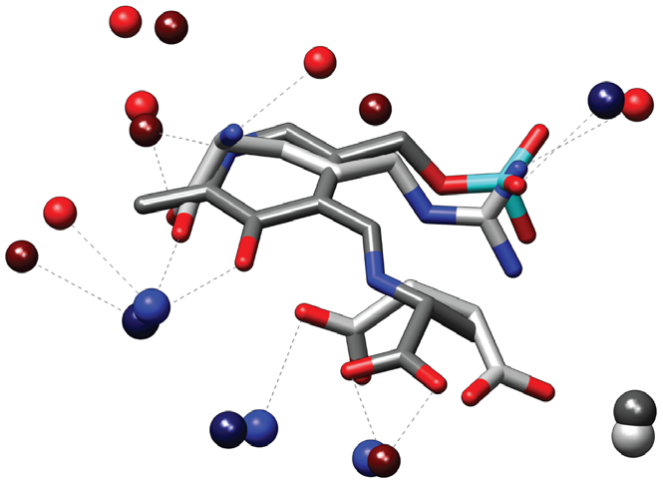
Superimposed ligands and matching pseudoatoms. The ligands of serine racemase from *Schizosaccharomyces pombe* (2zpu) and Argininosuccinate synthetase from *Thermus thermophilus* (1kor) superimposed according to the binding site similarity identified by superpose3D. This residue description uses pseudoatoms representing specific side-chain groups. The matching pseudoatoms are shown as spheres. 2zpu is shown with darker colors.

A further example of the usefulness of this residue representation is shown in Figure 7, which depicts the similarity between the binding sites of Serine racemase from *Schizosaccharomyces pombe* (2zpu) [32] and Argininosuccinate synthetase from *Thermus thermophilus* (1kor) [33]. These proteins belong to different PFAM [34] families and have very low sequence identity (20%). They also bind completely different ligands. Argininosuccinate synthetase is complexed with arginine and succinate while serine racemase is covalently bound with a modified Pyridoxal phosphate moiety (PLP-D-Ala). Superpose3D identifies a similarity comprising nine pseudoatoms belonging to eight residues with an RMSD of 0.64 Å. Interestingly the structural match overlays the ligands so that arginine and succinate are superimposed with different parts of the PLP-D-Ala molecule (see Figure 8). Arginine is superimposed to the pyridoxal phosphate moiety, with the guanidinium group in the same position as the phosphate of PLP. Succinate occupies a position corresponding to the alanine moiety of PLP-D-Ala. Again the identification of this similarity was possible because a residue description focused on chemical groups was used. For instance Tyr84 of the Argininosuccinate synthetase and Ser308 of the Serine racemase, which are both hydrogen bonded to their respective ligands, only have their terminal oxydrile superimposed while the remaining atoms occupy completely different positions.

### Assessing the significance of the results

The problem of assessing the statistical significance of local structural similarities has not been definitely solved yet. A number of methods have been proposed [35]–[38] often resulting in models which are parametrized by fitting to the results of comparisons between random pairs of structures. Unfortunately it is unclear whether the values of these parameters still hold when different methods are used or when the same method is applied to real-world datasets that can have all kinds of different biases. Moreover no score takes into account the geometry of the residues as they are only dependent on variables such as the size of the match, the RMSD etc. However a match of six residues located, for instance, in two alpha-helices is relatively common. On the other hand a match of the same size between residues in two binding sites could be extremely significant.

We therefore propose the following guidelines to interpret the results of superpose3D:

1. If the desired outcome of the analysis is a limited number of best matches from a database of several structures then it suffices to pick the longest matches, which are usually a handful against a large background of very small matches. RMSD is less significant since the software will try to extend the matches until they are just below the threshold.
2. The target dataset should be carefully chosen. For instance if one is interested in the study of binding sites it does not make sense to compare the entire structures. Similarly these programs should not be applied to structures which are globally similar.
3. If possible orthogonal criteria should be used to validate the results. These may include the position of bound ligands (if any), whether the residues of one of the two proteins are part of a known functional site etc., depending on the specific application at hand.
4. If one wants to have an idea of how uncommon a given pattern is the best thing is to search for it in a non-redundant sample of unrelated structures to derive an empirical distribution.

In order to show how these guidelines can be applied to a real-world case we discuss the results of using the residues comprising the p-loop of H-RAS (PDB code 5p21) as probes to scan a database of protein structures. We used a culled version of the PDB downloaded from the PISCES website [39]. This dataset includes 18534 chains which were derived from the PDB by selecting only structures with a resolution of 3.0 Angstroms or better and R-factor less than 1.0, and then clustering the proteins at the 90% sequence identity level. We used superpose3D with a representation including the C-α and the geometric centroid of the side chain and retained all the residues of the target chains.

We obtained 49710 matches 48556 (98%) of which comprise only three residues. These three-residue matches clearly represent the background noise and can be discarded, also considering that the p-loop comprises nine residues. To validate the remaining matches we used the following simple criterion: since the p-loop binds phosphate a match is considered significant if any residue is located close to a phosphorous atom (less than 4.5 Angstroms).

We therefore sorted the 1154 remaining matches (49710 - 48556) in decreasing size order first and then in increasing RMSD order for those of the same size. One way to assess whether this ordering correlates with our definition of significance is to use the ranking to predict which matches are significant and calculate the area under the ROC curve (AUC). If the ordering perfectly reflected the significance the AUC would be 1, meaning that there is a point in the ranking that perfectly separates significant from non-significant matches. We obtained an AUC of 0.87 which is a very high value and shows that these simple criteria are effective in locating the most promising matches in a database screening.

Moreover of the 10 top scoring matches that do not have a phosphate bound all the eight that are present in the SCOP classification belong to the p-loop containing nucleoside triphosphate hydrolases fold. Therefore such matches are clearly significant but the ligand is missing from the structure.

### Availability

We have developed the most flexible method available for local structural comparison. The usefulness of having a general-purpose software was demonstrated with several examples. superpose3D (available at http://cbm.bio.uniroma2.it/superpose3D) is fully open source and is the only structural comparison software that runs on Windows, Mac OSX and Linux.
